# Efficacy of nebulized GM-CSF inhalation in preventing oral mucositis in patients undergoing hematopoietic stem cell transplantation: A retrospective study

**DOI:** 10.1016/j.heliyon.2024.e37721

**Published:** 2024-09-12

**Authors:** Fenglian Luo, Li Zhao, Qi Zhang, Yunyun Yuan, Jun Cai

**Affiliations:** Department of Hematology, The Second Affiliated Hospital of Chongqing Medical University, No.288, Tian-Wen Road, Chongqing, 40000, China

**Keywords:** Oral mucositis, Granulocyte-macrophage colony-stimulating factor (GM-CSF), Nebulization, Hematopoietic stem cell transplantation (HSCT)

## Abstract

**Objective:**

To study the efficacy of oxygen atomization inhalation of granulocyte-macrophage colony-stimulating factor (GM-CSF) for preventing oral mucositis in patients following hematopoietic stem cell transplantation.

**Methods:**

Data from patients who received hematopoietic stem cell transplantation and were treated with GM-CSF for the prevention/treatment of oral mucositis in our hospital from June 2021 to June 2023 were collected. The enrolled patients were divided into an observation group and a control group according to the use of GM-CSF. The WHO Mucositis Scale Assessment Criteria were utilized to evaluate the characteristics of patients with oral mucositis (OM) from the beginning of the pretreatment period until they were discharged from the hospital. The general data, preconditioning protocol, transplantation method, overall grade and duration of oral mucositis, pain score, nutritional score and number of days of parenteral nutrition use, oral mucosal infection status and antibiotic use intensity, the granulocyte and megakaryocyte reconstruction time, and adverse reaction reports of the patients were collected and summarized through the medical records system.

**Results:**

A total of 143 patients were included in this study, including 75 patients in the observation group. In the observation group, there were 36 males and 39 females aged 22–67 years. There were 45 patients who received autologous transplantation and 30 patients who received allogeneic transplantation. In terms of the disease distribution, there were 33 cases of leukemia, 24 cases of lymphoma, 11 cases of multiple myeloma, and 8 other cases (3 cases of aplastic anemia, 2 cases of myelodysplastic syndrome, 2 cases of myelofibrosis, 1 case of POEMS syndrome). There were 68 patients in the control group, including 33 males and 35 females; the control group patients were aged 25–74years. Forty-one patients received autologous transplantation, and 27 patients received allogeneic transplantation. The disease distribution included 29 cases of leukemia, 17 cases of lymphoma, 12 cases of multiple myeloma, and 7 other cases (3 cases of aplastic anemia, 2 cases of myelodysplastic syndrome, 1 case of myelofibrosis, 1 case of POEMS syndrome). There were no significant differences between the two groups concerning age, sex, disease distribution or the transplantation method (P > 0.05). In the observation group, 13 cases did not develop oral mucositis, and 32 cases developed oral mucositis (16 cases of Grade I, 14 cases of Grade II, 2 cases of Grade III, and 0 cases of Grade IV). In the control group, there were 5 cases without mucositis and 36 cases with oral mucositis (6 cases of Grade Ⅰ, 16 cases of Grade Ⅱ, 8 cases of Grade Ⅲ, and 6 cases of Grade Ⅳ), the difference was statistically significant (P < 0.05). The pain score and duration of mucositis in the observation group were significantly lower than those in the control group (P < 0.05). In addition, the oral infection rate, antibiotic use intensity, nutritional score, per capita number of days of parenteral nutrition use and hematopoietic reconstruction time in the observation group were significantly lower than those in the control group (P < 0.05). In the observation group, 8 patients did not develop oral mucositis, and 22 patients developed oral mucositis (13 cases of Grade I, 7 cases of Grade II, 1 case of Grade III, and 1 case of Grade IV). In the control group, 1 case did not develop mucositis, and 26 cases developed oral mucositis (3 cases of Grade Ⅰ, 10 cases of Grade Ⅱ, 9 cases of Grade Ⅲ, and 4 cases of Grade Ⅳ). The difference was statistically significant (P < 0.05). The pain score and duration of mucositis in the observation group were significantly lower than those in the control group (P < 0.05). In addition, the oral mucosal infection rate, antibiotic use intensity, nutritional score, per capita number of days of parenteral nutrition use and hematopoietic reconstruction time in the observation group were significantly lower than those in the control group (P < 0.05). No adverse reactions were reported in either group.

**Conclusion:**

In both autologous transplantation and allogeneic transplantation patients, GM-CSF atomized inhalation can improve the prevention and treatment of oral mucositis in stem cell transplantation patients, reduce the incidence of oral infection, reduce the intensity of antibiotic use and the number of days of parenteral nutrition use, and thus promote the process of hematopoietic reconstruction.

## Highlights

• This study collected data from patients who underwent hematopoietic stem cell transplantation at our hospital from June 2021 to June 2023 and received GM-CSF for the prevention or treatment of oral mucositis. Based on GM-CSF utilization, these enrolled patients were divided into an observation group and a control group. The WHO Mucositis Scale Assessment Criteria were used to evaluate oral mucositis characteristics from pre-treatment until discharge. General patient data, pre-conditioning, transplantation methods, overall grade and duration of oral mucositis, pain scores, nutritional scores, days of parenteral nutrition use, oral mucosal infection, antibiotic use intensity, granulocyte and megakaryocyte reconstruction times, and adverse reaction reports were collected and summarized through the medical record system. Whether in autologous or allogeneic transplantation patients, GM-CSF atomized inhalation improved the prevention and treatment of oral mucositis, reduced oral infection incidence, decreased antibiotic use intensity, and shortened parenteral nutrition use days, thus promoting hematopoietic reconstruction.

## 1 Introduction

Hematopoietic stem cell transplantation (HSCT) is an effective treatment for many benign and malignant hematological diseases [1]. Oral mucositis (OM) is one of the most common toxic side effects in patients treated with HSCT and usually occurs 3–10 days after transplantation^[^ [2,3]^]^ Due to the toxic effects of pretreatment chemotherapy, radiotherapy and drugs before transplantation, submucosal endothelial cells, mucosal epithelial cells and connective tissue can be harmed, thus causing symptoms of oral mucositis in patients [1].

In individuals receiving hematopoietic stem cell transplantation, the prevalence of oral mucositis is high, ranging between 60 and 85 %. This prevalence is notably more pronounced, at 87 %, in those undergoing autologous hematopoietic stem cell transplantation. In particular, this higher incidence is associated with the use of camostin, etoposide, cytarabine, and melphalan in the conditioning regimens, as indicated by previous studies [4,5]. Severe oral mucositis can lead to total parenteral nutrition (TPN) use, opioid use, increased hospital stays, increased medical costs, and even increased mortality [6,7].

Oral mucositis causes damage to the integrity of the mucosal barrier, creating conditions for pathogens and inflammatory mediators to enter the bloodstream and increasing the chance of systemic infection [8]. Once ulcers form, the risk of systemic infections such as sepsis, bacteremia, and fungi is further increased in patients with neutropenia [9,10].

In clinical practice, managing oral mucositis involves a diverse array of intricate approaches. These include cryotherapy, agents that protect against radiation and chemicals, a variety of mouthwashes (both commercially produced and homemade), ointments, sprays, and traditional Chinese medicinal remedies. Nonetheless, the efficacy of these methods shows considerable variation, and a standardized protocol for prevention and treatment has not been established [11,12]. Efforts to assess different medications and approaches for the prevention or treatment of oral mucositis have been numerous, but the outcomes of these studies frequently present conflicting findings [13].

Granulocyte-macrophage colony-stimulating factor (GM-CSF) is a multipotent myeloid growth factor that promotes mucosal healing through mechanisms such as stimulating the production of wound healing factors from myeloid mononuclear-derived macrophages, encouraging endothelial cell migration and proliferation, inducing keratinocytes into a regenerative state, activating fibroblasts, and promoting granulation tissue formation [[14], [15], [16], [17]]. Previous studies have shown that the local use of GM-CSF can effectively control the occurrence of oral mucositis, reduce the severity of mucositis reactions, alleviate pain, shorten the duration of pain, and accelerate the healing of mucositis [[18], [19], [20]]. However, Harris et al. suggested that different routes of administration and patient compliance may affect treatment outcomes [21]. Furthermore, there are few reports on the use of nebulized GM-CSF for preventing oral mucositis. Its efficacy, potential adverse reactions during use, differences in effects on autologous and allogeneic transplant patients, and overall impact on stem cell transplant recipients remain unclear.

In this retrospective study, the researchers compared GM-CSF nebulization and GM-CSF gargling in autologous and allogeneic transplantation patients and their effects on patients to explore more effective strategies to prevent and treat oral mucositis. The researchers hope that this study will provide a valuable reference for clinicians when choosing more effective treatment options.

## 2 Patients and methods

The current study involved a retrospective analysis of stem cell transplantation patients treated with GM-CSF for oral mucositis at our hospital from June 2021 to June 2023. Patients were excluded based on the following specific criteria: (1) patients where GM-CSF was not employed for either the prevention or treatment of oral mucositis; (2) patients with incomplete data records; and (3) patients whose oral mucositis was a result of graft-versus-host disease (GVHD). In this study, 7 patients in the observation group and 11 patients in the control group were excluded due to incomplete data.

The Ethics Committee of the Second Affiliated Hospital of Chongqing Medical University approved this study (1.0/2023.11.1).

### 2.1 Patient

From June 2021 to June 2023, our hospital admitted 143 patients who underwent stem cell transplantation and received GM-CSF therapy for oral mucositis. Within this cohort, the observation group consisted of 75 patients, including 36 males and 39 females. The patients ranged in age from 22 to 67 years; the median age was 51 (22–67) years. In this group, there were 45 instances of autologous transplantation and 30 instances of allogeneic transplantation. The types of diseases present in this group varied, including 33 cases of leukemia, 24 cases of lymphoma, 11 cases of multiple myeloma, and 8 cases of other conditions (3 cases of aplastic anemia, 2 cases of myelodysplastic syndrome, 2 cases of myelofibrosis, and 1 case of POEMS syndrome). The control group was composed of 68 patients, including 33 males and 35 females. Their ages ranged from 25 to 74 years; the median age was 48(25–74) years. Within this group, 41 individuals underwent autologous transplantation, while 27 underwent allogeneic transplantation. The distribution of diseases in this group included 29 cases of leukemia, 17 cases of lymphoma, 12 cases of multiple myeloma, and 7 cases of other conditions, including 3 cases of aplastic anemia, 2 cases of myelodysplastic syndrome, 1 case of myelofibrosis, and 1 case of POEMS syndrome. A comparative analysis of the two groups revealed no significant differences in terms of age, sex, diagnosis, conditioning regimens, or type of transplantation, as indicated by a P value greater than 0.05; these findings are further elaborated in Table 1.

**Table 1:** Patient's demographics characteristics.

Variable	Observation group (n = 75)	Control group(n = 68)	*P value*
Gender			*P = 0.062*
Male	27	35	
Female	48	33	
Age(years)	51(22–67)	48(25–74)	*P = 0.735*
Type of malignancy			*P = 0.233*
Leukemia	33	29	
Lymphoma	24	21	
Multiple myeloma	11	12	
others	7	6	
Type of transplantation			*P = 0.971*
Autologous	45	41	
Allogeneic	30	27	
Conditioning regimens			*P = 0.406*
Melphalan	10	8	
Etoposide + Cyclophosphamide+ Busulfan	9	10	
Cyclophosphamide + Busulfan	11	9	
Cyclophosphamide + Busulfan + Cytarabine	23	22	
Fludarabine + Busulfan + Cytarabine	22	19	

### 2.2 Treatment

The conditioning regimens included both autologous transplantation and allogeneic transplantation. All patients received supportive care, which included antibiotics, antifungal and antiviral medications, prophylaxis for pneumocystis jirovecii pneumonia, proton pump inhibitors, standard antiemetics, and vitamin/electrolyte supplementation. Pain relief medication was administered promptly when oral mycosis pain affected eating and rest. Antibiotics were added when infection symptoms appeared. In HLA-identical sibling HSCT, the routine immunosuppressants used were cyclosporine and methotrexate. For haploidentical transplant patients, the immunosuppressants used posttransplant were cyclophosphamide (PTCy) and tacrolimus. When patients experienced severe mucositis with significant pain affecting eating and oral intake, and if the oral intake was less than 50 % of the daily requirement, total parenteral nutrition (TPN), including lipid emulsion and amino acids, was initiated [22]. To prevent oral mucositis, both groups routinely used a 3 % sodium bicarbonate mouthwash solution four times daily from the first day of conditioning. GM-CSF (300 μg of GM-CSF in 0.9 % saline solution, 15–20 ml each time, swished for 10–15 min, four times daily) was added to the 3 % sodium bicarbonate mouthwash solution. In contrast, in the observation group, in addition to the 3 % sodium bicarbonate mouthwash, GM-CSF oxygen nebulization (150 μg of GM-CSF in 10 ml of 0.9 % saline solution, nebulized with high-flow oxygen at 8 L per minute, twice daily) was used until hematopoietic reconstitution or oral mucositis healing, as detailed in Fig. S1.

### 2.3 Data collection

The following data were collected from the hospital's medical records: general patient information (sex age, diagnosis), conditioning regimens, transplantation method, antibiotic intensity, duration of parenteral nutrition (TPN) use, time to neutrophil and platelet reconstitution, and any adverse reactions. Oral mucosal swabs and cultures were collected to assess the presence of oral infections. The patients' oral mucositis grading, duration, and pain scores were gathered from nursing records. All data were collected from the start of pretreatment until patient discharge, excluding patients with OM and TPN caused by graft-versus-host disease (GVHD).

### 2.4 Oral mucositis evaluation criteria

The severity of oral mucositis in the study was classified following the criteria set by the 10.13039/100004423World Health Organization (10.13039/100004423WHO) as follows: Grade 0: normal; Grade I: erythema and mild pain, no interference with eating; Grade II: erythema, pain, edema, ulcers, and the ability to consume soft foods; Grade III: erythema, pain, edema, ulcers, and the ability to consume only a liquid diet; and Grade IV: unable to eat, requiring parenteral/enteral nutritional support [23]. Grades I-II were considered mild to moderate, while Grades III-IV were categorized as severe [24,25].

### 2.5 Pain assessment criteria

To assess the intensity of pain experienced by patients, the Numeric Rating Scale (NRS) was utilized. This scale ranges from 0 to 10, with 0 denoting no pain and 10 indicating extremely severe, unbearable pain [23].

### 2.6 Hematopoietic reconstitution evaluation criteria

The criteria for granulocyte recovery were met when the first occurrence of a consecutive three-day period achieved a neutrophil count ≥0.5 × 10^9/L. Platelet recovery was defined as the first occurrence of a consecutive three-day period with a platelet count ≥20 × 10^9/L, occurring within 7 days without platelet transfusion [26,27].

### 2.7 Nutritional scoring criteria

The Nutritional Risk Screening 2002 (NRS2002) was used to assess the nutritional status of the patients. This assessment tool derives its total score from both the nutritional evaluation and disease severity and has been evaluated and validated in multiple studies, demonstrating its reliability. An 10.13039/100003186NRS score of <3 points indicate no risk of malnutrition, whereas an 10.13039/100003186NRS score of ≥3 points suggest a high risk of malnutrition or significant malnutrition, indicating the need for nutritional support [23].

### 2.8 Oral infection assessment criteria

Oral infection status was determined based on the examination and culture results of oral mucosal smears once a week. A positive result indicated the presence of an infection, while a negative result indicated the absence of an infection.

### 2.9 Statistical methods

Two individuals were responsible for entering the data into the database, and the statistical analysis was conducted using SPSS 26.0 software. Continuous variables are expressed as median (range). The recovery times for granulocytes and megakaryocytes were analyzed using independent samples t tests, whereas Welch's *t*-test was applied to evaluate the nutritional scoring and the duration of parenteral nutrition usage. Categorical data are presented as n/percentage. The intensity of antibiotic usage was analyzed using the chi-squared (χ^2^) test. Oral infection status was assessed using Yates-corrected chi-squared tests, and the occurrence and grade of oral mucositis were determined using Fisher's exact test, with statistical significance set at P < 0.05.

## 3 Results

### 3.1 The incidences of OM, pain score, and duration of oral mucositis

**Fig. 1:**
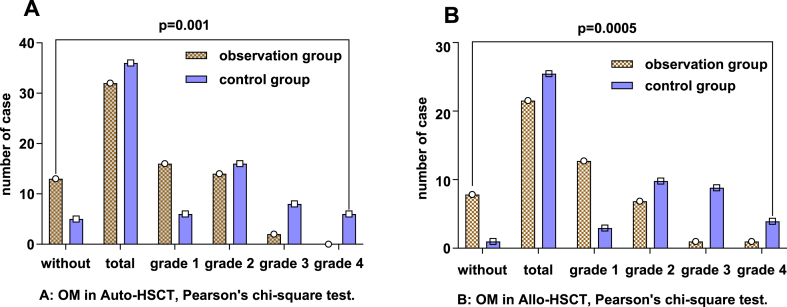
The severity of oral mucositis in patients who underwent auto-PBSCT and Allo-HSC transplantation. **A:** Evaluation of the cases of without, grade 1, grade 2, grade 3, grade 4, and total OM in the observation group (n = 45) and the control group (n = 41) after Auto-HSCT. **B:** Comparison of without, grade 1, grade 2, grade 3, grade 4, and total OM cases in the observation group (n = 30) and the control group (n = 27) after Allo-HSCT. as determined by Pearson chi-square test.

**Fig. 2:**
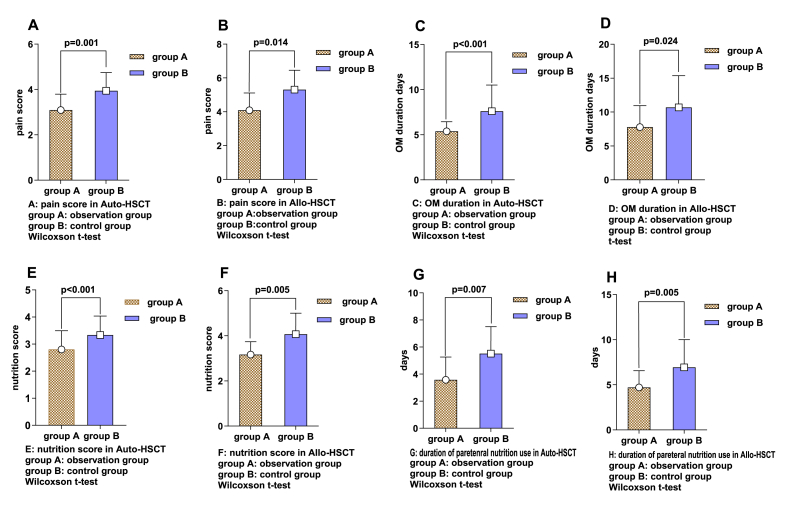
Pain score, duration of OM, nutrition score, duration of parenteral nutrition uses in the observation group and the control group following auto-PBSCT and Allo-HSC transplantation **A-D:** Comparison between the two groups of pain score and OM duration after auto-PBSCT **(A, C)** and Allo-HSCT **(B, D)** in the observation group (n=45) and the control group (n=41). **E-H:** Comparison between the two groups of nutrition score and the duration of parenteral nutrition use following auto-PBSCT **(E, G)** and Allo-HSCT **(F, H)** in the observation group (n=30) and the control group (n=27). Wilcoxson t-test is used in A-C, and E-H to identify which group differences are significant. At the same time, an independent samples t-test was used in D.

Among the autologous transplant patients, the observation group had 13 cases without oral mucositis and 32 cases with oral mucositis, with 16 cases categorized as Grade I, 14 cases categorized as Grade II, 2 cases categorized as Grade III, and no cases categorized as Grade IV. In contrast, the control group included 5 patients without mucositis and 36 patients with oral mucositis, with 6 cases categorized as Grade I, 16 cases categorized as Grade II, 8 cases categorized as Grade III, and 6 cases categorized as Grade IV. The disparity between these groups was statistically significant, with a P-value less than 0.05(Fig. 1A). Patients in the observation group reported a pain score of (3.094 ± 0.777), and the duration of their oral mucositis averaged (5.375 ± 1.070) days. Conversely, in the control group, the pain score was higher at (3.944 ± 1.264), with oral mucositis lasting longer, averaging (7.611 ± 2.901) days. Both the duration of oral mucositis and the pain scores were significantly lower in the observation group compared to those in the control group, as indicated by a P-value less than 0.05(Fig. 2A and C). Among the allogeneic transplant patients in the observation group, 8 cases (26.67 %) had no oral mucositis, while 22 cases developed oral mucositis, with 13 cases categorized as Grade I, 7 cases categorized as Grade II, 1 case categorized as Grade III, and 1 case categorized as Grade IV. In the control group of allogeneic transplant patients, there was 1 case without mucositis, while 26 cases experienced oral mucositis, with 3 cases categorized as Grade I, 10 cases categorized as Grade II, 9 cases categorized as Grade III, and 4 cases categorized as Grade IV. This difference was statistically significant, with a P value less than 0.05(Fig. 1B). For the observation group, the pain score was (4.091 ± 1.411), and the average duration of oral mucositis was (7.773 ± 3.167) days. In comparison, the control group reported a greater pain score (5.308 ± 2.074) and a longer duration of oral mucositis (10.692 ± 4.671) days (Fig. 2B and D). Both the duration of oral mucositis and the pain scores were significantly lower in the observation group than in the control group, as indicated by the P value.

### 3.2 Nutritional score and duration of parenteral nutrition use

Among the patients who received autologous transplants, those in the observation group had a nutritional score of 2.800 ± 0.694 and duration of parenteral nutrition use for an average of (3.578 ± 2.598) days. In contrast, the control group had a nutritional score of (3.366 ± 0.698), with an average duration of parenteral nutrition usage of (5.512 ± 3.376) days. The observation group had significantly lower nutritional scores than did the control group (P < 0.05) (Fig. 2E and G). For the allogeneic transplant patients, the nutritional score for the patients in the observation group was (3.167 ± 0.791), and the duration of parenteral nutrition usage was (4.700 ± 2.231) days, while in the control group, the nutritional score was (4.074 ± 1.412), and the duration of parenteral nutrition usage was (6.926 ± 3.316) days. The nutritional scores in the observation group were significantly lower than those in the control group, as indicated by a P value less than 0.05 (Fig. 2F and H), indicating statistical significance.

### 3.3 The incidence of oral mucosal infection and antibiotic usage

**Fig. 3:**
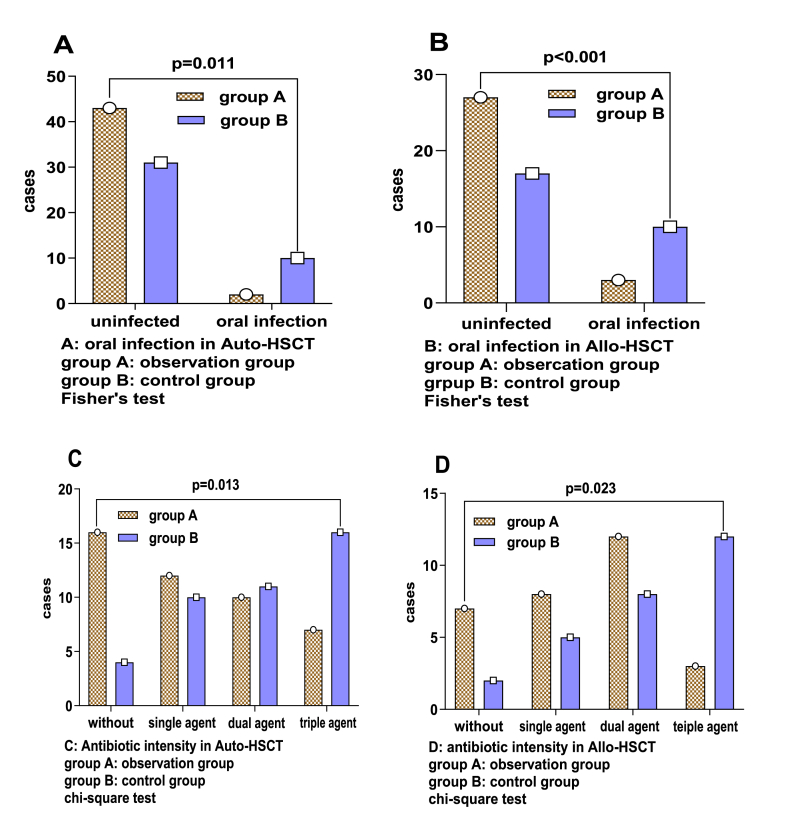
The incidence of oral mucosal infection and antibiotic usage in the observation group and the control group following auto-PBSCT and Allo-HSC transplantation. **A-B**: oral infection following auto-PBSCT **(A)** and Allo-HSCT **(B)** are compared between the observation (n=45) and control groups (n=41), Fisher's exact test is utilized to ascertain significant group differences. **C-D:** The use of antibiotics—categorized as none, single-agent, dual-agent, and triple-agent—after auto-PBSCT **(C)** and Allo-HSCT**(D)** was compared between the observation group (n=30) and the control group (n=27). Pearson chi-square test is utilized to ascertain significant group differences.

In the autologous transplant patients, the incidence of oral mucosal infection in the observation group was 4.44 % (2/45), and the antibiotic usage intensity was as follows: not used, 35.55 % (16/45); single agent, 26.67 % (12/45); dual agent, 22.22 % (10/45); and triple agent, 15.56 % (7/45). In the control group, the incidence of oral mucosal infection was 24.39 % (10/41), and the antibiotic usage intensity was as follows: not used, 9.76 % (4/41); single agent, 24.39 % (10/41); dual agent, 26.83 % (11/41); and triple agent, 39.02 % (16/41). The observation group had lower incidences of oral mucosal infection compared to the control group (P < 0.05) (Fig. 3A and C), with statistically significant differences. In the allogeneic transplant patients, the incidence of oral mucosal infection in the observation group was 10 % (3/30), and the antibiotic usage intensity was as follows: not used, 23.33 % (7/30); single agent, 26.67 % (8/30); dual agent, 40.00 % (12/30); and triple agent, 10.00 % (3/30). In the control group, the incidence of oral mucosal infection was 37.4 % (10/27), and the antibiotic usage intensity was as follows: not used, 7.41 % (2/27); single agent, 18.52 % (5/27); dual agent, 29.63 % (8/27); and triple agent, 44.44 % (12/27). The observation group had lower incidences of oral mucosal infection compared to the control group (P < 0.05) (Fig. 3B and D), with statistically significant differences.

### 3.4 Granulocyte and Platelet reconstitution

**Fig. 4:**
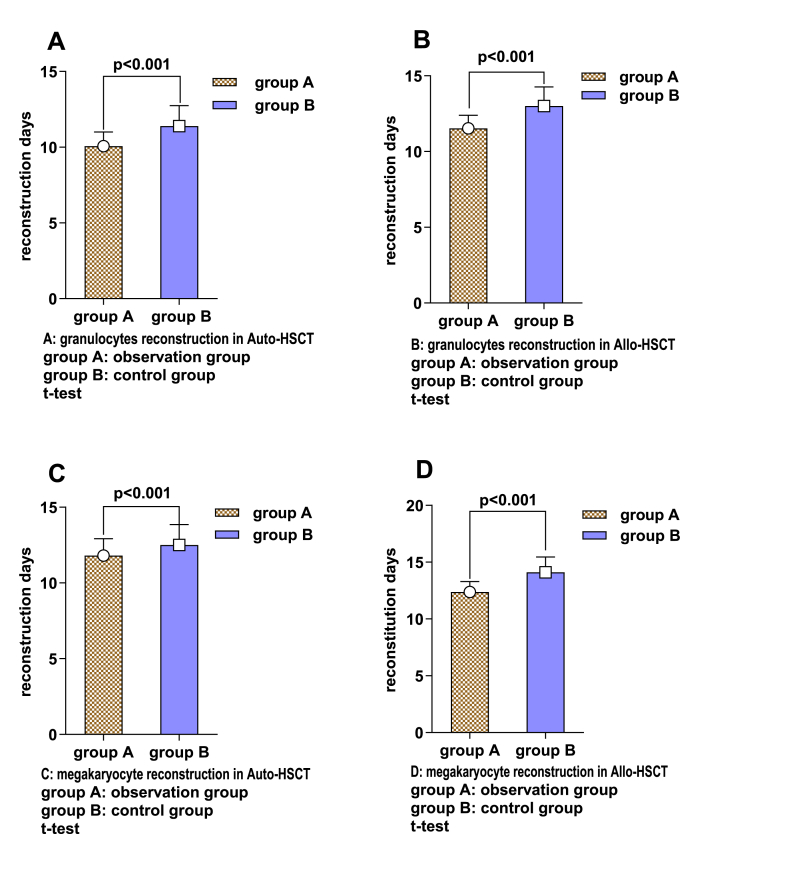
Granulocyte and Platelet reconstitution in the observation group and the control group after auto-PBSCT and Allo-HSC transplantation. **A-B**: Comparison between the two groups of granulocytes reconstruction after auto-PBSCT (**A**) and allo-PBSCT (B) in the observation group (n=45) and the control group (n=41). **C-D**: The median time needed for megakaryocyte reconstruction following allo-PBSCT**(C)** and allo-PBSCT **(D)** between the observation group (n=30) and the control group (n=27). Data are presented as mean ± SD (standard deviation). Independent samples t-test is used to identify which group differences are significant.

In the autologous transplant patients, the time to granulocyte recovery for patients in the observation group was (10.067 ± 0.939), and the time to platelet recovery was (11.089 ± 1.104), while in the control group, the time to granulocyte recovery was (11.390 ± 1.358), and the time to platelet recovery was (12.512 ± 1.344). The observation group had significantly shorter recovery times than did the control group (P < 0.05) (Fig. 4A and C). In the allogeneic transplant patients, the time to granulocyte recovery for patients in the observation group was (11.533 ± 0.860), and the time to platelet recovery was (12.367 ± 0.928), while in the control group, the time to granulocyte recovery was (13.00 ± 1.271), and the time to platelet recovery was (14.111 ± 1.340). The observation group had significantly shorter recovery times than did the control group (P < 0.05) (Fig. 4B and D). In both the autologous and allogeneic transplant patients, no adverse reactions were reported in either group.

## 4 Discussion

In this study, the researchers found that among patients who underwent autologous or allogeneic transplantation, most individuals in the observation group experienced mild to moderate oral mucositis. Conversely, in the control group, oral mucositis was predominantly severe. This indicates that the administration of GM-CSF through inhalation in a nebulized form is more effective in preventing and treating oral mucositis than the administration of GM-CSF via mouthwash. Although clinical reports indicate that GM-CSF mouthwash is currently the most effective method for both the prevention and treatment of oral mucositis [28], its practical application often presents challenges. This is because patient compliance can vary, affecting the duration and correctness of mouth rinsing, which directly impacts the amount of time the medication has sufficient contact with the oral mucosa. As a result, this situation may impact, to some degree, the progression and severity of oral mucositis (OM), along with the therapeutic effectiveness of the treatment [29,30].

In this study, the principle of atomizing GM-CSF involves high-speed oxygen airflow passing through the capillary mouth of the atomizer to generate negative pressure at the tube mouth. This negative pressure sucks the drug liquid out from the adjacent tube mouth and causes it to transform into tiny droplets at the capillary mouth, which are then ejected into the patient's mouth in the form of an aerosol. The drug can be delivered directly to the mucosal surface of the mouth, thereby maximizing exposure to the site of inflammation. This local delivery mode helps to improve the bioavailability of the drug, thus enhancing the therapeutic effect. Since 150 μg of GM-CSF were added to 10 ml of 0.9 % sodium chloride solution for injection for each patient, atomization was carried out at an oxygen flow rate of 8 L/min, which ensured that the atomization dose and atomization time were the same for each patient, thus ensuring the consistency of drug efficacy.

On the one hand, since GM-CSF is a protein preparation, its solution loses activity very easily at room temperature. To ensure its efficacy, the drug must be stored in a refrigerator at 2–8 °C for no more than 24 h. On the other hand, this increases the nursing workload, and when patient compliance is poor, also causes drug waste. In this study, patients were administered GM-CSF oxygen atomization b. i.d., and the drugs could be used on the spot. The remaining half of the drug preparation was stored in a refrigerator at 4 °C for use in the afternoon, which reduced drug waste. By analyzing data on adverse reactions, it was determined that no adverse reactions were observed during the administration of the drug. This suggests the relative safety of the medication, a finding that aligns with the research conducted by Philip Riley et al. [31].

In this study, the oral mucositis in the observation group had a shorter duration and was less severe in both the autologous and allogeneic transplantation patients. This finding may be because when GM-CSF atomized inhalation was used, the drug was in higher concentration when it made oral contact with the mucous membrane than the gargle form, and the effect of the drug on oral mucositis is dose-dependent. That is, the drug concentration is positively correlated with the effect [32]. Furthermore, administering the medication through high-flow oxygen nebulization allows for the medication to be dispersed as a mist or micro mist, which, when inhaled through the mouth, takes advantage of the patient's natural respiratory processes. This promotes the even distribution of the aerosol particles throughout various parts of the oral cavity, enhancing local drug absorption.

Additionally, providing oxygen to ulcerated areas can alleviate the hypoxia caused by ischemia in the oral mucosa, thereby reducing the impact of oral mucositis during the period of neutrophil depletion following HSCT. This, in turn, accelerates cell regeneration, contributing to the healing of mucositis. This finding is supported by research from Gordon and colleagues [33,34].

Oral mucositis is the most prevalent adverse effect among patients undergoing stem cell transplantation. It often results in pain and challenges eating and can act as a portal for infections. Oral Mucositis can lead to weight loss and an increased risk of secondary infections. These complications significantly complicate treatment, resulting in prolonged antibiotic use, extended hospital stays, increased treatment-related costs, and a reduced quality of life for patients [35,36]. The researchers also found that the oral infection rate and antibiotic use intensity in the observation group were significantly lower than those in the control group, mainly because the inhalation administration of drugs can directly act on the inflammation site, effectively reducing the occurrence of infection and thus shortening the duration of antibiotic use. Oral mucositis frequently manifests as intense pain, which impacts the patient's ability to eat and their overall quality of life. Particularly for patients with Grade IV oral mucositis, severe nutritional deficiencies are common, necessitating the reliance on parenteral nutrition to sustain the patient's nutritional status [37].

Importantly, in the process of autologous transplantation and allogeneic transplantation, the pain score, nutrition score, and days of parenteral nutrition use were significantly lower for the patients in the observation group than for the patient in the control group, mainly because atomized inhalation of GM-CSF reduced the patients’ oral mucositis severity and pain and improved their eating and chewing function. This reduces the need for and cost of parenteral nutrition. In terms of hematopoietic reconstruction, the reconstruction time of the granulocytes and megakaryocytes of the observation groups was lower than that of the control group, mainly because the patients in the observation group generally had milder oral mucositis and relatively lower pain scores; thus, they could eat effectively, which improved the intake of nutrients, effectively supplementing the nutrients required for cell growth in the body and promoting hematopoietic reconstruction to a certain extent.

## 5 Conclusion

Nebulized GM-CSF inhalation could effectively enhance the prevention and treatment of oral mucositis (OM) in patients following autologous or allogeneic transplant. This regimen will significantly diminish the risk of developing oral infections and alleviate oral pain. Concurrently, the use of nebulized GM-CSF also reduces the intensity of antibiotic usage and the duration of parenteral nutrition and further promotes hematopoietic recovery.

Although the results of this study were satisfactory, its limitations as a single-center retrospective study may limit the general applicability of the findings. In the future, we plan to conduct a multicenter randomized controlled trial (RCT) to ensure the broader applicability of the study results.

## Data availability

Data included in article/supp. material/referenced in the article is available.

## Funding

The authors declare that no funds, grants, or other support were received during the preparation of this manuscript.

## Ethics approval

This retrospective study was approved by Ethics Committee of the Second Affiliated Hospital of Chongqing Medical University (1.0/2023.11.1).

## CRediT authorship contribution statement

**Fenglian Luo:** Writing – review & editing, Writing – original draft, Project administration, Methodology, Investigation, Formal analysis, Data curation, Conceptualization. **Li Zhao:** Investigation, Data curation. **Qi Zhang:** Investigation, Data curation. **Yunyun Yuan:** Investigation, Data curation. **Jun Cai:** Supervision, Resources, Project administration, Conceptualization.

## Declaration of competing interest

The authors declare that they have no known competing financial interests or personal relationships that could have appeared to influence the work reported in this paper.
