# Supplementary material for: Efficacy of nebulized GM-CSF inhalation in preventing oral mucositis in patients undergoing hematopoietic stem cell transplantation: A retrospective study
Source: Heliyon. 2024 Sep 12;10(19):e37721. doi: 10.1016/j.heliyon.2024.e37721 (PMC11466551; doi:10.1016/j.heliyon.2024.e37721)
Supplement: Multimedia component 1 [file mmc1.docx]

Flow chart 1 Flow diagram of the study

**AHSCT**

**supportive treatment**

**conditioning regimen**

**Prevention of oral mucositis**

**GM-CSF**

**Mouthwash**

**GM-CSF**

**nebulization**

**TPN**

**Allo-HSCT**

**Haploid transplantation**

**Identical transplantation**

**supportive treatment**

**conditioning regimen**

**Prevention of oral mucositis**

**GM-CSF**

**Mouthwash**

**GM-CSF**

**nebulization**

**TPN**

**anti-infection**

**anti-infection**

**HSCT**
